# Efficacy of Dupilumab in a Woman With Severe Asthma Complicated by Eosinophilic Sinusitis and a Desire to Have a Baby: A Case Report

**DOI:** 10.1002/ccr3.70982

**Published:** 2025-10-08

**Authors:** Akari Neoi, Masakiyo Yatomi, Yurino Nagata, Daisuke Higeta, Daisuke Shimizu, Karin Kuno, Yuri Sawada, Mari Sato, Haruka Aoki‐Saito, Koichi Yamaguchi, Hiroaki Tsurumaki, Yosuke Miura, Kenichiro Hara, Noriaki Sunaga, Yasuhiko Koga, Kazuaki Chikamatsu, Takeshi Hisada, Toshitaka Maeno

**Affiliations:** ^1^ Division of Allergy and Respiratory Medicine, Integrative Center of Internal Medicine Gunma University Hospital Maebashi Japan; ^2^ Medical Corporation Sanshikai Toho Hospital Midorishi Japan; ^3^ Department of Otolaryngology‐Head and Neck Surgery Gunma University Graduate School of Medicine Maebashi Japan; ^4^ Department of Obstetrics and Gynecology Gunma University Hospital Maebashi Japan; ^5^ Department of Rehabilitation Sciences Gunma University Graduate School of Health Sciences Maebashi Japan

**Keywords:** dupilumab, eosinophilic sinusitis, infertility treatment, pregnant women, severe asthma

## Abstract

Dupilumab is an effective biologic therapy for severe asthma; however, its safety and efficacy in women undergoing infertility treatment remain uncertain. A woman in her late 30s with severe asthma and eosinophilic chronic rhinosinusitis underwent assisted reproductive technology while receiving dupilumab. The medication was discontinued upon confirmation of pregnancy. Asthma control improved significantly with dupilumab. The patient conceived successfully and delivered a healthy infant. Dupilumab may be a viable treatment option for women with severe asthma undergoing fertility treatment, though additional safety data are needed to support its use during conception and pregnancy.

## Graphical abstract

Since 2008, the patient had been undergoing asthma treatment, but it was poorly controlled. She also had comorbid conditions including eosinophilic sinusitis, allergic rhinitis, conjunctivitis, and a non‐functional adrenal tumor. After marriage in 2018 (age 34 years), her asthma control remained poor, and she was unable to conceive spontaneously. Infertility treatment was pursued but was unsuccessful. However, after switching dupilumab in June 2022, her asthma control improved, and she successfully conceived via assisted reproductive technology. After confirming pregnancy, dupilumab was discontinued, and treatment was adjusted by increasing inhaled medications. She gave birth to a healthy infant in September 2024.

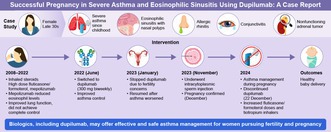

**Summary**

- A woman in her late 30s, treated with dupilumab for severe asthma associated with eosinophilic sinusitis and infertility, became pregnant and gave birth via Assisted Reproductive Technology.
- Dupilumab was administered prior to pregnancy. It may be considered for young women with eosinophilic sinusitis and severe asthma seeking pregnancy.

## 1 Introduction

Asthma is primarily caused by lower respiratory tract type II inflammation, which accounts for more than 80% of asthma cases [1]. Poor asthma control decreases quality of life and often requires oral corticosteroids (OCS), which can lead to infections and other side effects. Severe asthma, which accounts for 7%–10% of cases, has benefited greatly from the recent introduction of biological agents. Asthma is often complicated by upper airway allergic diseases, and the concept of a united airway disease has been proposed [2]. Allergic rhinitis and chronic rhinosinusitis are involved in asthma development and poor control [2], and airway remodeling resulting from type II inflammation and tissue damage is common [3].

Mepolizumab is an anti‐interleukin‐5 (IL‐5) monoclonal antibody product that became available for treating severe asthma in Japan in 2016. Eosinophils and IL‐5 are involved in asthma development, and anti‐IL‐5 antibody treatment reduces the frequency of exacerbations and OCS dose [4]. Dupilumab is an IL‐4α/IL‐13α receptor antibody product that inactivates IL‐4 and IL‐13 and suppresses type II inflammation, leading to reduced asthma exacerbations and improved respiratory function [5]. In recent years, dupilumab has proven effective for severe asthma as well as atopic dermatitis and eosinophilic sinusitis [6, 7].

The efficacy and safety of dupilumab administration have been reported in pregnant women with atopic dermatitis [8]; however, there is a lack of knowledge regarding its administration to pregnant women with severe asthma or eosinophilic sinusitis, or during infertility treatment.

We report a case of a woman with severe asthma complicated by eosinophilic sinusitis who had improved asthma control and successfully conceived and delivered a baby via assisted reproductive technology (ART) with intracytoplasmic sperm injection (ICSI) after treatment with dupilumab. We evaluated the efficacy of dupilumab administration in this patient.

## 2 Case History/Examination

**FIGURE 1:**
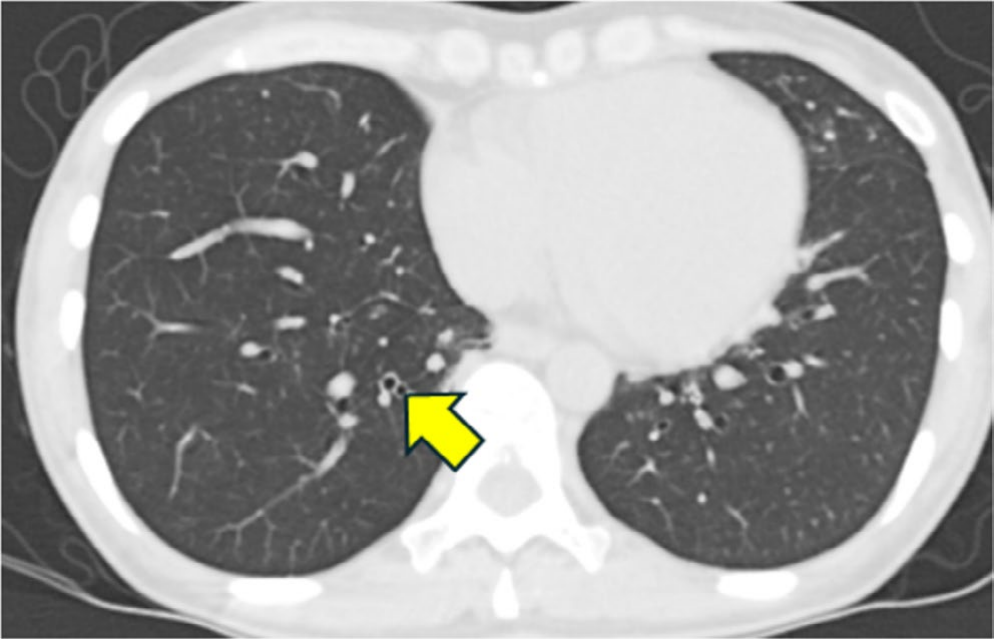
CT image taken on September 29, 2017, prior to administration of mepolizumab, showing bronchial wall thickening in the lower airway.

The patient had developed bronchial asthma during childhood and experienced recurrent asthma exacerbations during adulthood. She had no smoking history or complications of atopic dermatitis and had a body mass index of 16.3 kg/m^2^; however, she had allergic rhinitis, allergic conjunctivitis, and a nonfunctioning left adrenal tumor. At age 24 years, in June 2008, she started treatment with inhaled steroids. The percentage of the forced expiratory volume in the first second decreased to 79.9% at 29 years of age (Table 1). The patient was prescribed high doses of fluticasone propionate/formoterol fumarate hydrate (1000 μg/40 μg/day), tiotropium bromide hydrate (5 μg/day), and montelukast sodium (10 mg/day). Treatment with a fluticasone furoate nasal spray (110 μg/day) was also initiated, but the patient still experienced asthma exacerbations, mainly during the winter. Chest computed tomography (CT) revealed bronchial wall thickening, suggesting chronic airway inflammation (Figure 1). Olfactory disturbances were prominent, and a sinus biopsy performed on April 18, 2018, led to a diagnosis of eosinophilic sinusitis with few nasal polyps. An increased eosinophil count of 470/mL and IgE level of 320 IU/mL were noted on July 20, 2018.

**TABLE 1:** Changes in lung function.

	4/24/2012	1/9/2014	4/12/2021	5/18/2022	12/20/2022
VC (L)	3.05	2.89	2.71	2.79	2.75
%VC (%)	104.1	99.7	86.8	90.6	89.3
IC (L)	1.24	1.74	1.6	1.9	1.83
FVC (L)	3.09	2.94	2.64	2.74	2.72
FEV_1_ (L)	2.49	2.22	2.09	2.26	2.22
%FEV_1_ (%)	88	79.9	80.6	88.3	86.7
FEV_1. 0% (Gensnar)_ (%)	80.58	75.51	79.16	82.48	81.62

*Note:* Lung function at age 28 on April 24, 2012, was good in terms of VC and FEV1.0. Lung function before mepolizumab at 30 years of age on January 9, 2014, showed respiratory dysfunction with a%FEV1.0 < 80%. Lung function at 37 years of age on April 12, 2021, more than 2 years after mepolizumab administration, showed the lowest FEV1.0. Lung function before dupilumab administration at 38 years of age on May 18, 2022, showed an increase in FEV1.0 and %FEV1.0. Lung function 6 months after dupilumab administration at the age of 38 years on December 20, 2022, showed no significant change compared to lung function before dupilumab administration.

Abbreviations: %FEV1.0, percent forced expiratory volume in 1 s; %VC, percent vital capacity; FEV1.0, forced expiratory volume in 1 s; FVC, forced vital capacity; IC, inspiratory capacity; VC, vital capacity.

## 3 Differential Diagnosis, Investigations, and Treatment

**FIGURE 2:**
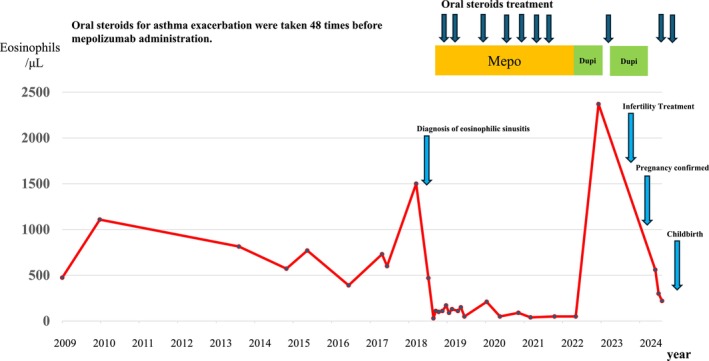
The peripheral blood eosinophil count was high from the first visit, with repeated asthma exacerbations and a sharp increase in eosinophil count in 2018. After mepolizumab administration in July 2018, the eosinophil count fell and the frequency of asthma exacerbations decreased. After switching to dupilumab in June 2022, the eosinophil count increased because of side effects of dupilumab. Dupilumab treatment was discontinued after pregnancy was discovered. Dupi, dupilumab; Mepo, mepolizumab.

**FIGURE 3:**
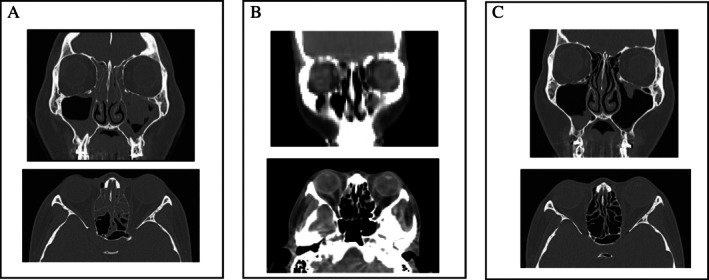
Sinus CT image taken on March 24, 2018, prior to mepolizumab administration, showing findings of severe sinusitis, mainly in the ethmoidal sinus region. (A) PET‐CT image taken on April 24, 2020, more than 1 year after starting mepolizumab, showing marked improvement in sinusitis. (B) Sinus CT image taken on August 17, 2022, 2 months after starting dupilumab, showing no change compared to B and no evidence of recurrent sinusitis.

The patient's physician had recommended omalizumab since 2016, but the patient declined because of concerns about its potential lack of effectiveness. In 2018, as signs of type II inflammation became increasingly evident, the physician proposed mepolizumab. The patient agreed, and treatment with subcutaneous mepolizumab (100 mg every 4 weeks) was initiated. This led to a rapid reduction in eosinophil count (Figure 2). Positron emission tomography–CT, performed to investigate a coexisting adrenal tumor, revealed marked improvement in sinusitis (Figure 3). Furthermore, pulmonary function improved following the initiation of mepolizumab (Table 1). However, asthma exacerbations continued to occur once or twice a year.

On June 12, 2022, treatment was switched from mepolizumab to subcutaneous dupilumab (300 mg every 2 weeks). As shown in Figure 2, the eosinophil count increased 6 months later—a known side effect of dupilumab; however, the Asthma Control Test (ACT) score improved from 16 to 20, and asthma exacerbations ceased. Thus, the inhaled dose of fluticasone propionate/formoterol fumarate hydrate was reduced (500 μg/20 μg/day). Furthermore, the Lund‐Mackay score remained low, and her sense of smell recovered mildly (Table 2).

**TABLE 2:** Changes in the Lund‐Mackay score on sinus CT.

Sinus	3/26/2018		4/24/2020		8/17/2022	
	Left	Right	Left	Right	Left	Right
Maxillary (0, 1, 2)	1	1	1	1	1	1
Anterior ethmoid (0, 1, 2)	2	2	0	1	0	0
Posterior ethmoid (0, 1, 2)	1	1	0	0	0	0
Sphenoid (0, 1, 2)	0	1	0	0	0	0
Frontal (0, 1, 2)	1	1	0	0	0	0
OMC (0, 2)	2	2	0	0	0	0
Total	7	8	1	2	1	1
0: No abnormality 1: Partial opacification 2: Total opacification						
OMC: Ostiomeatal complex 0: Non‐obstructed 2: Obstructed						

*Note:* The Lund‐Mackay score on sinus CT prior to mepolizumab administration on March 26, 2018, was elevated, a finding suggestive of severe sinusitis. Sinus positron emission tomography‐CT 1 year and 9 months after mepolizumab administration on April 24, 2020, showed a marked decrease in Lund‐Mackay score. A sinus CT on August 17, 2022, 2 months after dupilumab administration, showed that the Lund‐Mackay score remained low.

The patient wished to get pregnant after her marriage in 2018 (age 34 years) but was unable to conceive spontaneously. Her condition improved after receiving dupilumab, and she was considered for fertility treatment at age 38 years. Dupilumab administration was stopped on January 6, 2023, considering its impact on the fetus. However, as asthma symptoms worsened, dupilumab treatment was restarted on February 24. Her symptoms improved, and she began ovulation induction with follitropin alfa on July 14. She subsequently underwent two intrauterine inseminations and one in vitro fertilization; however, after unsuccessful attempts, she was switched to ICSI. Following ICSI, a 5‐day‐old embryo was transferred on December 7, and pregnancy was confirmed on December 26 (at 39 years and 10 months of age). For perinatal safety, dupilumab was discontinued after the December 22 administration. Thereafter, the ACT score dropped to 18 points, and the patient experienced two asthma exacerbations, requiring OCS at 15 mg/day for several days. After pregnancy was detected, the patient was treated with an increased dose of fluticasone propionate/formoterol fumarate hydrate (1000 μg/40 μg/day), combined with tiotropium bromide hydrate inhalation. The fetus developed well, and on September 9, 2024, at 40 weeks, a baby (weight, 2874 g; length, 54 cm; Apgar score, 8 at 1 min and 9 at 5 min) was delivered. Ethical approval from the institutional review board of our university was not required for this case report.

## 4 Outcome

After switching from the administration of mepolizumab, dupilumab further reduced the frequency of asthma exacerbations and significantly improved the quality of life from infertility treatment to delivery.

## 5 Discussion

We report a case of a woman who desired to become pregnant after improving asthma control and successfully underwent fertility treatment while receiving dupilumab, resulting in a regular delivery.

The patient had Cluster II asthma according to the classification by Moore et al. [9] Moreover, when the April 2018 asthma treatment was considered, it corresponded to step 5 on the 2024 GINA severe asthma guide, and asthma treatment for type II inflammation was indicated [10]. Additionally, when the June 2022 asthma treatment strategy was reviewed in the Algorithm for the Assessment and Treatment of Adults with Uncontrolled Severe Asthma, omalizumab, dupilumab, and tezepelumab were more likely to be successful [11]. The eosinophil count before dupilumab administration was 50/μL as a result of the effect of mepolizumab, but the exhaled NO was 50 ppb, indicating that dupilumab treatment was expected to be successful.

Complicated eosinophilic sinusitis was also a key therapeutic factor in this case—dupilumab and mepolizumab have previously been successful in the treatment of severe asthma with eosinophilic sinusitis [12]; however, in this case, dupilumab was more effective in reducing asthma exacerbations. OCS was administered 48 times (4.8/year) prior to mepolizumab and seven times (1.79/year) during the mepolizumab treatment period; however, after switching to dupilumab, asthma exacerbations ceased. Nakagome et al. reported that switching to dupilumab is recommended for patients with severe asthma who do not respond to anti‐IL‐5 treatment and with FeNO levels > 25 ppb [13]. Notably, dupilumab reduces airway mucus secretion by suppressing IL‐13 signaling [14]. Increased IL‐4/IL‐13 levels have also been reported in respiratory tract infections [15]. Therefore, the inhibitory effect of dupilumab on IL‐4 and IL‐13 may reduce asthma exacerbations. Furthermore, Nopsopon et al. showed that dupilumab could have a stronger effect on asthma exacerbation than mepolizumab, which is consistent with the present case [16].

Regarding pregnancy in patients with asthma, complicated pregnancies may be associated with an increased risk of perinatal death, congenital anomalies, prematurity, low birth weight, and adverse maternal outcomes [17]. Asthma exacerbation, OCS use, and asthma severity are reported to be associated with preterm delivery, low birth weight, and fewer weeks of gestation [18]. Furthermore, poor asthma control has been reported to induce menstrual irregularities and infertility [19]. In the present case, the patient was on OCS until the start of dupilumab treatment.

This patient was an underweight woman, and with regard to fertility, a previous report has shown that time to conception is prolonged in women with a body mass index of < 18.5 [20]. In this case, the woman's low body weight, in addition to her age, may have contributed to her infertility. In 2022, the Japan Society of Obstetrics and Gynecology ART Clinical Practice document stated that the pregnancy rate/total treatment rate for women aged 39 years was 26.9%, the production rate/total treatment was 17.9%, and the miscarriage rate/total pregnancy was 30.3%. In the present case, the patient succeeded in conceiving relatively early after beginning infertility treatment, and fetal development was favorable until delivery. However, the patient experienced worsening asthma symptoms and sinusitis after discontinuation of dupilumab, which may have increased the risk of worsening fetal growth.

The patient received 11 doses of dupilumab from the start of infertility treatment until pregnancy. However, the safety of dupilumab in the perinatal period could not be adequately confirmed since it was discontinued after pregnancy was detected. Omalizumab is currently the only biological treatment for severe asthma that is safe for pregnant women [21]. It is crucial to investigate the safety profile of biologics during pregnancy and infertility treatment. In this case, the results suggest that dupilumab administration during fertility treatment did not cause any abnormalities in the oocytes or fertilized eggs. Good outcomes have also been reported in pregnant women with atopic dermatitis who were treated with dupilumab during pregnancy, as well as in their fetuses [22]. Furthermore, the safety of dupilumab has been reported in a pregnant woman with atopic dermatitis complicated by asthma [23]. In contrast, this case demonstrated the efficacy of dupilumab during infertility treatment, pregnancy, and delivery in a patient with predominantly severe asthma without atopic dermatitis.

In the present case, although mepolizumab had some effect, dupilumab further reduced the frequency of asthma exacerbations and significantly improved the quality of life from infertility treatment through to delivery. However, as dupilumab was administered only before pregnancy was confirmed, there are limitations to discussing its safety during pregnancy and infertility treatment. The potential benefits and risks of dupilumab to the fetus should be carefully evaluated, and further safety data are needed. This case suggests that biologics may be considered early in the treatment of young female patients with severe asthma, and that dupilumab may play a central role in managing women with severe asthma complicated by eosinophilic sinusitis who wish to conceive. This case report may contribute to the advancement of asthma treatment in women seeking to become pregnant.

## Author Contributions

**Akari Neoi:** conceptualization, data curation, formal analysis, investigation, methodology, resources, validation, writing – original draft, writing – review and editing. **Masakiyo Yatomi:** conceptualization, data curation, formal analysis, investigation, methodology, project administration, resources, validation, visualization, writing – original draft, writing – review and editing. **Yurino Nagata:** conceptualization, data curation, formal analysis, investigation, resources, validation, visualization, writing – original draft. **Daisuke Higeta:** conceptualization, data curation, formal analysis, investigation, methodology, resources, validation, writing – review and editing. **Daisuke Shimizu:** data curation, formal analysis, investigation, methodology, validation. **Karin Kuno:** data curation, formal analysis, investigation, methodology, resources. **Yuri Sawada:** data curation, formal analysis, investigation, methodology, resources. **Mari Sato:** data curation, formal analysis, investigation, methodology, resources. **Haruka Aoki‐Saito:** data curation, formal analysis, investigation, methodology, resources. **Koichi Yamaguchi:** data curation, formal analysis, investigation, methodology, resources. **Hiroaki Tsurumaki:** conceptualization, formal analysis, investigation, supervision, validation, writing – review and editing. **Yosuke Miura:** conceptualization, formal analysis, investigation, supervision, validation, writing – review and editing. **Kenichiro Hara:** conceptualization, formal analysis, investigation, supervision, validation, writing – review and editing. **Noriaki Sunaga:** conceptualization, formal analysis, investigation, supervision, validation, writing – review and editing. **Yasuhiko Koga:** conceptualization, formal analysis, investigation, supervision, validation, writing – review and editing. **Kazuaki Chikamatsu:** conceptualization, formal analysis, investigation, supervision, validation, writing – review and editing. **Takeshi Hisada:** conceptualization, formal analysis, investigation, supervision, validation, writing – review and editing. **Toshitaka Maeno:** conceptualization, formal analysis, investigation, supervision, validation, writing – review and editing.

## Disclosure

Provenance and peer review: This article was not commissioned and was peer‐reviewed.

## Consent

The patient provided detailed informed written consent for the incorporation of her medical history, images, laboratory reports, and personal information into the case report.

## Conflicts of Interest

Hiroaki Tsurumaki has conflicts of interest regarding speaking fees from Sanofi and GlaxoSmithKline. Takeshi Hisada has a conflict of interest regarding speaking fees from GlaxoSmithKline. The other authors have no conflicts of interest.

## Data Availability

The data that support the findings of this study are available from the corresponding author upon reasonable request.
